# Is lymph node metastasis an advanced event of gastrointestinal stromal tumor?

**DOI:** 10.1093/oncolo/oyaf351

**Published:** 2025-10-17

**Authors:** Qi Jiang, Peng Zhang, Weili Yang, Xiaodong Gao, Yingfeng Fu, Jun Zhang, Bo Zhang, Fan Feng, Gang Zhai, Yang Fu, Xin Wu, Xinhua Zhang, Xiaojun Wu, Zhidong Gao, Han Liang, Yanbing Zhou, Heli Liu, Kaixiong Tao

**Affiliations:** Department of Gastrointestinal Surgery, Union Hospital, Tongji Medical College, Huazhong University of Science and Technology, Wuhan 430022, China; Department of Gastrointestinal Surgery, Union Hospital, Tongji Medical College, Huazhong University of Science and Technology, Wuhan 430022, China; Department of Gastrointestinal Surgery, The First Affiliated Hospital, Zhejiang University School of Medicine, Hangzhou 310003, China; Department of General Surgery, Zhongshan Hospital, Fudan University, Shanghai 200032, China; Department of Gastrointestinal Surgery, Taihe Hospital of Hubei University of Medicine, Shiyan 442000, China; Department of Gastrointestinal Surgery, The First Affiliated Hospital of Chongqing Medical University, Chongqing 400016, China; Department of Gastrointestinal Surgery, West China Hospital, Sichuan University, Chengdu 610041, China; Division of Digestive Surgery, Xijing Hospital of Digestive Diseases, The Air Force Medical University, Xi’an 710032, China; Department of General Surgery, Shanxi Province Cancer Hospital (Shanxi Cancer institute), Taiyuan 030013, China; Department of Gastrointestinal Surgery, The First Affiliated Hospital of Zhengzhou University, Zhengzhou, 450052, China; Department of General Surgical Medicine, The First Medicine Center of PLA General Hospital, Beijing 100853, China; Department of Gastrointestinal Surgery, The First Affiliated Hospital, Sun Yat-sen University, Guangzhou 510080, China; State Key Laboratory of Oncology in South China, Collaborative Innovation Center for Cancer Medicine, Department of Colorectal Surgery, Sun Yat-sen University Cancer Center, Guangzhou 510060, China; Department of Gastrointestinal Surgery, Peking University People’s Hospital, Peking University, Beijing 100044, China; Department of Gastric Cancer Center, Tianjin Medical University, Cancer Institute & Hospital, Tianjin 300060, China; Department of Gastrointestinal Surgery, Affiliated Hospital of Qingdao University, Qingdao 266003, China; Department of Gastrointestinal Surgery, Xiangya Hospital, Central South University, Changsha, Hunan 410008, China; Department of Gastrointestinal Surgery, Union Hospital, Tongji Medical College, Huazhong University of Science and Technology, Wuhan 430022, China

**Keywords:** gastrointestinal stromal tumor, lymph node metastasis, propensity score matching, outcome

## Abstract

**Objective:**

The clinicopathological features and outcome of gastrointestinal stromal tumors (GISTs) with lymph node metastasis remain controversial owing to their low incidence. A multicenter retrospective cohort study was conducted to compare the clinicopathological features and oncologic outcomes of GIST without metastasis, with lymph node metastasis, and with distant metastasis.

**Design:**

The medical records of patients with GISTs in 16 large medical centers in China from January 2014 to December 2022 were reviewed. Patients were divided into four groups: no metastasis (988 cases, 89.1%), lymph node metastasis without distant metastasis (75 cases, 6.8%), distant metastasis without lymph node metastasis (36 cases, 3.2%), and distant metastasis with lymph node metastasis group (10 cases, 0.9%). Propensity score matching (PSM) was performed to reduce confounding factors.

**Result:**

A total of 1109 cases of primary GIST were included in this study, comprising 607 males (54.7%) and 502 females (45.3%), with a mean age of 56.6 ± 11.9 years. Compared to that in GIST without lymph node metastasis, the proportion of nongastric GIST was higher in GIST with lymph node metastasis (52.9% vs 40.7%) with a larger tumor diameter (>10 cm: 36.5% vs 18.1%) and more patients with distant metastasis (11.8% vs 3.5%). Tumor location not in the stomach, the largest tumor diameter, and distant metastasis were independent risk factors for GIST with lymph node metastasis (all *P *< .05). After PSM, 96, 48, 24 patients comprised no metastasis, lymph node metastasis without distant metastasis, and distant metastasis without lymph node metastasis, respectively. The relapse-free survival (RFS) of the lymph node metastasis group was comparable to that of the distant metastasis group without lymph node metastasis (*P *= .368) and significantly inferior to that of no metastases (*P *= .042).

**Conclusions and Relevance:**

The RFS of patients with GIST with lymph node metastasis was comparable to those with distant metastasis and significantly worse than those without metastasis. Lymph node metastasis is an advanced event in GIST.

Implications for Practice The clinicopathological features and outcome of gastrointestinal stromal tumors (GISTs) with lymph node metastasis remain controversial owing to their low incidence. This multicenter retrospective cohort study analyzed the data of 1109 patients with primary GIST and found that tumor location, tumor diameter, and distant metastasis were independent risk factors for lymph node metastasis in GIST. Patients with lymph node metastasis exhibited significantly inferior RFS compared to those without metastasis, and comparable RFS to those with distant metastasis. These findings suggest that lymph node metastasis is an advanced event in GIST.

## Introduction

Gastrointestinal stromal tumors (GISTs) are the most common gastrointestinal mesenchymal tumors, and the stomach is the most common site of the disease, followed by the small intestine, rectum, and esophagus. A small number of gastrointestinal stromal tumors can also occur outside the gastrointestinal tract.^1^ In contrast to gastrointestinal epithelial tumors, GIST metastases are commonly observed with both hepatic and intraperitoneal dissemination. Among patients with recurrence and metastasis of GISTs, liver metastasis accounts for 66.7%, intra-abdominal spread accounts for approximately 50.0%, and lymph node metastasis is extremely rare.^2^

Since the American Joint Committee on Cancer (AJCC) published the TNM staging of GIST for the first time in 2009, it regards lymph node metastasis as an important parameter that reflects the biological behavior of GIST and affects the outcome: GIST is stage IV if lymph node metastasis occurs.^3^ However, few clinical studies have supported GIST lymph node metastasis as an advanced event. The aim of this study, which included retrospectively collected GIST data from lymph node dissection in 16 large tertiary-grade centers in China, was to analyze the clinicopathological characteristics of GIST lymph node metastasis and to establish the first predictive model for accurately predicting GIST lymph node metastasis. In addition, propensity score matching (PSM) was used to reduce confounding factors among the lymph node metastasis group, the group with distant metastasis, and the group without metastasis, and the relapse-free survival (RFS) of the three groups was compared to provide a reference for the precise treatment of GIST.

## Methods

### Study population

**Figure 1.**
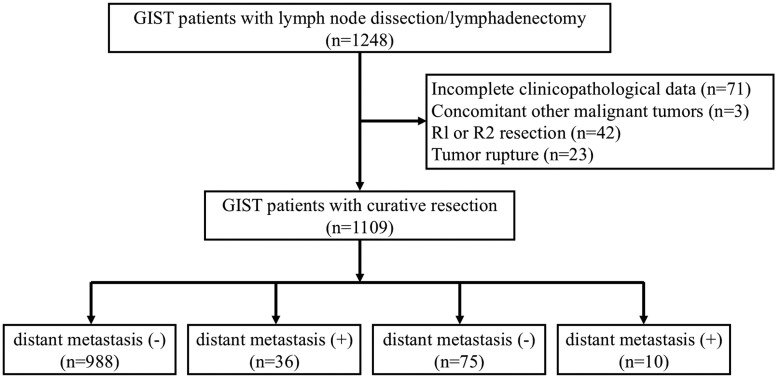
Study flowchart. Patients with gastrointestinal stromal tumors (GISTs) underwent lymph node dissection from January 2014 to December 2020.

This was a multicenter, real-world, retrospective study. The inclusion criteria were as follows: (1) complete surgical resection; (2) evaluation of lymph node status by preoperative imaging and intraoperative evaluation; (3) lymph node dissection or lymphadenectomy (targeted removal of specific lymph nodes) for lymphadenopathy; (4) postoperative pathological diagnosis of primary GIST; and (5) the status of lymph node and the presence or absence of distant metastasis documented by postoperative pathological report. Exclusion criteria were: (1) incomplete clinicopathological data, (2) association with other malignant tumors, (3) tumor rupture, and (4) perioperative death. Based on the above criteria, the clinicopathological data of patients with GIST who received surgical treatment in 16 large tertiary hospitals in China from January 1, 2014, to December 31, 2022, were collected. The case inclusion process is shown in Figure 1. This study was approved by the Ethics Committee of Union Hospital, Tongji Medical College, Huazhong University of Science and Technology (approval number: 20230523), which confirmed that informed consent was not necessary.

### Observed indicators

Clinicopathological information, such as on age, sex, preoperative treatment, tumor location, tumor size, mitotic count, histological type, tumor necrosis or cystic degeneration, recurrence and metastasis, and adjuvant therapy, was collected. According to the National Institutes of Health (NIH) risk classification criteria, the risk classification of GIST is divided into very low-, low-, intermediate-, and high-risk groups, based on tumor size, mitotic index, primary tumor site, and tumor rupture.^4^ RFS was defined as the time from surgery to first tumor recurrence and metastasis.

### Patient follow-up

According to the patient’s risk grading, within 2 years after surgery, re-examination was conducted every 3 months; from 3 to 5 years after surgery, the patients were reviewed every 6 months; and after 5 years, they were reviewed every year. The patients were followed up by telephone, outpatient clinic visits, and an online platform every 3-6 months, and the last follow-up date was December 2021. The follow-up included investigating the survival and tumor recurrence of patients and their status concerning their receiving targeted drug therapy.

### Statistical methods

Statistical analysis was performed using SPSS 22.0 and R 4.03 software. Quantitative data conforming to a normal distribution are expressed as the mean ± standard deviation. Categorical data are expressed as cases (%), and the chi-square test or Fisher’s exact test was used for comparisons between groups. Survival curves were drawn using the Kaplan–Meier method. The log-rank test was used for univariable prognostic analysis, and the Cox proportional hazards model was used for multivariable prognostic analysis. The above tests were all two-sided, and *P *< .05 was considered to indicate statistically significant.

Patients were divided into four groups: no metastasis, lymph node metastasis without distant metastasis, distant metastasis without lymph node metastasis, and distant metastasis with lymph node metastasis group according to the pathological reports following complete resection. Due to the limited number of cases in the group with concurrent lymph node and distant metastases, this subgroup was excluded from subsequent prognostic analyses. The no metastasis, lymph node metastasis without distant metastasis, and distant metastasis without lymph node metastasis groups were compared using PSM. The balancing factors for PSM included age, sex, preoperative treatment, tumor location, tumor size, mitotic pattern, histological type, tumor necrosis or cystic degeneration, and postoperative adjuvant therapy. The PSM of distant metastasis without lymph node metastasis, lymph node metastasis without distant metastasis, and no metastasis groups was performed in a 1:2:4 ratio. A standardized difference of less than 10% was considered acceptable and used to eliminate the different distributions of covariates after matching.^5^

## Results

### Baseline characteristics of patients

In total, 1109 cases of primary GIST were included in this study, comprising 607 men (54.7%) and 502 women (45.3%), with a mean age of 56.6 ± 11.9 years. There were 1024 patients (92.3%) with no lymph node metastasis after surgery, and 85 patients (7.7%) had lymph node metastasis. Tumors were in the stomach of 647 patients (58.3%) and not in the stomach in 462 patients (41.7%). The histological types were spindle cells in 964 cases (86.9%), epithelioid in 30 cases (2.7%), and mixed cells in 115 cases (10.4%). A total of 301 patients (27.1%) had tumors with necrosis or cystic degeneration, and 46 (4.1%) had distant metastasis.

A total of 573 patients in the entire group underwent genetic testing, and 485 patients (84.6%) had *KIT* gene mutations, including 430 patients with *KIT* 11 mutations, 42 patients with *KIT* 9 mutations, 3 patients with *KIT* 13 mutations, and 2 patients with *KIT* 17 mutations. Four patients had *KIT* 11 and 13 double mutations, two had *KIT* 11 and 17 double mutations, and two had *KIT* 9 and 11 double mutations. Fifteen patients (2.6%) had *PDGFRA* mutations, of whom 12 had *PDGFRA* 18 mutations and 3 had *PDGFRA* 12 mutations. There were 73 cases (12.7%) of wild-type GISTs, of which 8 were SDHB-deficient GISTs. Genetic testing was performed on 45 patients in the lymph node metastasis group, and 33 patients (73.3%) had *KIT* gene mutations, including 25 patients with *KIT* 11 mutations and 9 patients with *KIT* 8 mutations; 12 patients (26.7%) had wild-type GIST, of whom 4 patients had SDHB-deficient GIST. Eighty-eight (7.9%) patients received preoperative therapy, and 508 (45.8%) patients received postoperative adjuvant therapy.

Compared to GIST without lymph node metastasis, the proportion of nongastric GIST was higher in GIST with lymph node metastasis (52.9% vs 40.7%, *P *= .028), which had the largest tumor diameter (>10 cm: 36.5% vs 18.1%, *P *< .001), a higher number of mitoses (>10/50 HPF: 24.7% vs 16.6%, *P *= .008), more tumor necrosis or cystic degeneration (37.6% vs 26.3%, *P *= .023), and more patients with distant metastasis (11.8% vs 3.5%, *P *< .001), as shown in Table 1.

**Table 1.** Demographic and clinicopathological factors of all the patients.

Characteristics	Total (*n* = 1109)	No lymph node metastasis (*n* = 1024)	Presence of lymph node metastasis (*n* = 85)	*P* value
**Age**				.539
** ≤60**	674 (60.8%)	625 (61.0%)	49 (57.6%)	
** >60**	435 (39.2%)	399 (39.0%)	36 (42.4%)	
**Sex**				.310
** Male**	607 (54.7)	556 (54.3%)	51 (60.0%)	
** Female**	502 (45.3%)	468 (45.7%)	34 (40.0%)	
**Preoperative therapy**				.915
** Yes**	88 (7.9%)	81 (7.9%)	7 (8.2%)	
** No**	1021 (92.1%)	943 (92.1%)	78 (91.8%)	
**Tumor site**				.028
** Stomach**	647 (58.3%)	607 (59.3%)	40 (47.1%)	
** Non-stomach**	462 (41.7%)	417 (40.7%)	45 (52.9%)	
**Tumor size (cm)**				<.001
** ≤2**	122 (11.0%)	119 (11.6%)	3 (3.5%)	
** 2-5**	346 (31.2%)	331 (32.3%)	15 (17.6%)	
** 5-10**	425 (38.3%)	389 (38.0%)	36 (42.4%)	
** >10**	216 (19.5%)	185 (18.1%)	31 (36.5%)	
**Mitotic count**				.008
** ≤5**	695 (62.7%)	655 (64.0%)	40 (47.1%)	
** 6-10**	223 (20.1%)	199 (19.4%)	24 (28.2%)	
** >10**	191 (17.2%)	170 (16.6%)	21 (24.7%)	
** Site histotype**				.069
** Spindle**	964 (86.9%)	897 (87.6%)	67 (78.8%)	
** Epithelioid**	30 (2.7%)	26 (2.5%)	4 (4.7%)	
** Mix**	115 (10.4%)	101 (9.9%)	14 (16.5%)	
**Tumor necrosis or cystic degeneration**				.023
** Yes**	301 (27.1%)	269 (26.3%)	32 (37.6%)	
** No**	808 (72.9%)	755 (73.7%)	53 (62.4%)	
**Distant metastasis**				<.001
** Yes**	46 (4.1%)	36 (3.5%)	10 (11.8%)	
** No**	1063 (95.9%)	988 (96.5%)	75 (88.2%)	
**Targeted therapy**				.110
** Yes**	508 (45.8%)	462 (45.1%)	46 (54.1%)	
** No**	601 (54.2%)	562 (54.9%)	39 (45.9%)	

### Selection and design of predictors for lymph node metastasis

**Figure 2.**
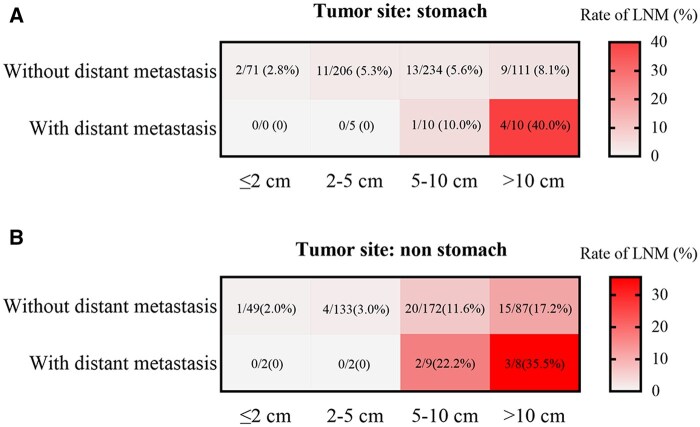
Incidence of lymph node metastasis (LNM) in patients with g-GISTs (gastrointestinal stromal tumors) and non-g GISTs. (A) LNM rates in g-GISTs with distant metastasis or without distant metastasis stratified by tumor size. (B) LNM rates in non-g GISTs with distant metastasis or without distant metastasis stratified by tumor size.

Multivariable logistic regression analysis showed that tumors not located in the stomach, the largest tumor diameter, and distant metastasis were independent risk factors for GIST with lymph node metastasis (all *P *< .05), as shown in Table 2. Furthermore, in this study, tumors were divided into gastric and nongastric GISTs, with lymph node metastasis as the *Y*-axis and tumor size as the *X*-axis, to construct a lymph node metastasis prediction heat map. The results showed that in patients with gastric GIST, the lymph node metastasis rate was 40.0% (4/10) in those with the largest tumor diameter >10 cm and distant metastasis. Among patients with nongastric GIST, the lymph node metastasis rate was 35.5% (3/8) in patients with tumors with the largest tumor diameter of >10 cm and distant metastasis, as shown in Figure 2.

**Table 2.** Logistic regression of factors associated with LNM in patients with GIST.

Variable	OR	(95% CI)	*P* value
**Tumor site**			
** Stomach**	Reference	-	-
** Non-stomach**	1.596	1.017-2.506	.042
**Tumor size (cm)**			
** ≤2**	Reference	-	-
** 2-5**	1.829	0.519-6.446	.347
** 5-10**	3.544	1.069-11.747	.039
** >10**	6.098	1.814-20.503	.003
**Distant metastasis**			
** No**	Reference	-	-
** Yes**	2.834	1.327-6.053	.007

### Oncologic outcomes

The median follow-up time for all patients was 45 months (range, 1–95 months). The 1- and 3-year RFS rates of the entire patient group were 98.8% and 95.6%, respectively. Univariable analysis showed that tumor location (*P *= .003), mitotic count (*P *< .001), histological type (*P *= .001), tumor necrosis or cystic degeneration (*P *= .005), lymph node metastasis (*P *< .001), distant metastasis (*P *< .001), and postoperative adjuvant therapy (*P *< .001) were significantly associated with patient outcome. Cox regression analysis (Table 3) showed that tumor location (*P *= .030), mitotic count (*P *= .045), histological type (*P *= .005), lymph node metastasis (*P *= .001), distant metastasis (*P *= .010), and postoperative adjuvant therapy (*P *< .001) were independent risk factors for RFS in all patients.

**Table 3.** Univariate and multivariate analyses of relapse-free survival analysis.

Characteristics	Univariate			Multivariate		
	HR	95% CI	*P* value	HR	95% CI	*P* value
**Age**						
** ≤60**						
** >60**	1.034	0.679-1.575	.876			
**Sex**						
** Female**						
** Male**	0.957	0.779-1.176	.677			
**Preoperative therapy**						
** Yes**						
** No**	1.616	0.811-3.217	.172			
**Tumor site**						
** Stomach**						
** Non-stomach**	1.876	1.244-2.831	.003	1.884	1.236-2.871	.030
**Tumor size (cm)**						
** ≤2**						
** 2-5**	0.528	0.208-1.342	.179	0.471	0.184-1.210	.111
** 5-10**	1.682	0.758-3.733	.201	0.930	0.404-2.145	.865
** >10**	2.075	0.905-4.755	.085	0.960	0.387-2.384	.930
**Mitotic count**						
** ≤5**						
** 6-10**	1.470	0.874-2.473	.146	1.324	0.757-2.317	.489
** >10**	2.393	1.482-3.864	<.001	1.752	1.011-3.036	.045
** Site histotype**						
** Spindle**						
** Epithelioid**	1.209	0.296-4.931	.791	1.483	0.357-6.150	.587
** Mix**	2.506	1.476-4.255	.001	2.198	1.271-3.801	.005
**Tumor necrosis or cystic degeneration**						
** No**						
** Yes**	1.826	1.201-2.776	.005	1.228	0.784-1.923	.370
**Lymph node metastasis**						
** No**						
** Yes**	3.552	2.182-5.781	<.001	2.398	1.440-3.992	.001
**Distant metastasis**						
** No**						
** Yes**	3.779	2.060-6.933	<.001	2.313	1.226-4.364	.010
**Targeted therapy**						
** No**						
** Yes**	0.308	0.197-0.481	<.001	0.420	0.265-0.664	<.001

### Subgroup analysis based on lymph node metastasis

**Figure 3.**
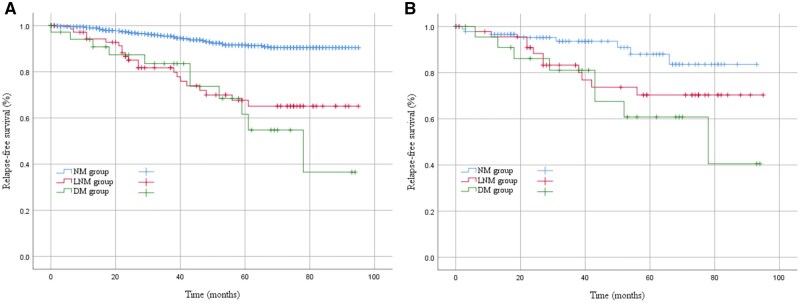
Comparison of relapse-free survival before (A) and after (B) propensity score matching (PSM) among no metastasis, lymph node metastasis without distant metastasis, and distant metastasis without lymph node metastasis groups.

The whole group of patients was divided into the no metastasis group (988 cases, 89.1%), lymph node metastasis without distant metastasis group (75 cases, 6.8%), distant metastasis without lymph node metastasis group (36 cases, 3.2%), and distant metastasis with lymph node metastasis group (10 cases, 0.9%). The propensity score was used to balance the baseline levels of the no metastasis, lymph node metastasis without distant metastasis, and distant metastasis without lymph node metastasis groups. After matching, the baseline levels of preoperative treatment, tumor size, mitotic count, and postoperative adjuvant therapy were all balanced among the three groups (all *P *> .05), as shown in Table 4. After PSM, the RFS of the lymph node metastasis without distant metastasis group was better than that of the no metastasis group (*P *= .042), while it was not significantly different from that of the distant metastasis without lymph node metastasis group (*P *= .368), as shown in Figure 3.

**Table 4.** Patient baseline clinical characteristics and surgical outcomes before and after propensity score-matching.

Characteristics	Before marching					After marching				
	No metastasis (*n* = 988, %)	lymph node metastasis without distant metsstasis (*n* = 75, %)	distant metsstasis without lymph node metastasis (*n* = 36, %)	*χ* *2/t*	*P*	No metastasis (*n* = 96, %)	lymph node metastasis without distant metsstasis (*n* = 48, %)	distant metsstasis without lymph node metastasis (*n* = 24, %)	*χ* *2/t*	*P*
**Age**				4.169	.124				0.828	.661
** ≤60**	608 (61.5)	41 (54.7)	17 (47.2)			49 (51.0)	22 (45.8)	10 (41.7)		
** >60**	380 (38.5)	34 (45.3)	19 (52.8)			47 (49.0)	26 (54.2)	14 (58.3)		
**Sex**				2.303	.316				0.483	.786
** Male**	533 (53.9)	45 (60.0)	23 (63.9)			63 (65.6)	30 (62.5)	14 (58.3)		
** Female**	455 (46.1)	30 (40.0)	13 (36.1)			33 (34.4)	18 (37.5)	10 (41.7)		
**Preoperative therapy**				15.258	<.001				0.261	.929
** Yes**	72 (7.3)	5 (6.7)	9 (25.0)			5 (5.2)	3 (6.3)	1 (4.2)		
** No**	916 (92.7)	70 (93.3)	27 (75.0)			91 (94.8)	45 (93.8)	23 (95.8)		
**Tumor site**				4.788	.091				0.447	.800
** Stomach**	587 (59.4)	35 (46.7)	20 (55.6)			51 (53.1)	24 (50.0)	11 (45.8)		
** Non-stomach**	401 (40.6)	40 (53.3)	16 (44.4)			45 (46.9)	24 (50.0)	13 (54.2)		
**Tumor size(cm)**				20.841	.002				3.794	.711
** ≤2**	117 (11.8)	3 (4.0)	2 (5.6)			7 (7.3)	2 (4.2)	1 (4.2)		
** 2-5**	324 (32.8)	15 (20.0)	7 (19.4)			15 (15.6)	8 (16.7)	3 (12.5)		
** 5-10**	373 (37.8)	33 (44.0)	16 (44.4)			33 (34.4)	21 (43.8)	13 (54.2)		
** >10**	174 (17.6)	24 (32.0)	11 (30.4)			41 (42.7)	17 (35.4)	7 (29.2)		
**Mitotic count**				13.87	.008				2.017	.733
** ≤5**	636 (64.4)	37 (49.3)	19 (52.8)			45 (46.9)	22 (45.8)	11 (45.8)		
** 6-10**	194 (19.6)	20 (26.7)	5 (13.9)			19 (19.8)	12 (25.0)	3 (12.5)		
** >10**	158 (16.0)	18 (24.0)	12 (33.3)			32 (33.3)	14 (29.2)	10 (41.7)		
** Site histotype**				9.293	.058				3.124	.521
** Spindle**	864 (87.4)	57 (76.0)	33 (91.7)			77 (80.2)	39 (81.3)	22 (91.7)		
** Epithelioid**	26 (2.6)	4 (5.3)	0 (0)			4 (4.2)	4 (8.3)	0 (0)		
** Mix**	98 (9.9)	14 (18.7)	3 (8.3)			15 (15.6)	5 (10.4)	2 (8.3)		
**Tumor necrosis or cystic degeneration**				5.282	.071				1.419	.492
** Yes**	257 (26.0)	28 (37.3)	12 (33.3)			28 (29.2)	16 (33.3)	10 (41.7)		
** No**	731 (74.0)	47 (62.7)	24 (66.7)			68 (70.8)	32 (66.7)	14 (58.3)		
**Targeted therapy**				12.386	.002				1.000	.607
** Yes**	436 (44.1)	39 (52.0)	26 (72.2)			48 (50.0)	22 (45.8)	14 (58.3)		
** No**	552 (55.9)	36 (48.0)	10 (27.8)			48 (50.0)	26 (54.2)	10 (41.7)		

## Discussion

GIST with lymph node metastasis is relatively rare, and previous small-sample studies have reported that the rate of GIST with lymph node metastasis was 5.1%-9.8%.^6–9^ In this study, clinicopathological data of patients with GIST with lymph nodes detected by pathology were collected from 16 large tertiary centers in China to investigate the clinicopathological features and outcome of GIST with lymph node metastasis.

Currently, the most widely used GIST risk grading standard worldwide is the modified NIH risk grading standard, which is mainly based on tumor size, mitotic count, tumor location, and tumor rupture. However, the relationship between lymph node metastasis and GIST risk remains unclear.^10^ Previous studies have found GIST tumors with lymph node metastasis were larger and had higher mitotic count.^8^^,^^9^ Our study also showed that the tumor size and mitotic count in the group with lymph node metastasis were higher than those in the group without lymph node metastasis, which is consistent with the literature. In addition, the proportion of tumor necrosis, cystic degeneration, and mixed cells in GIST patients with lymph node metastasis was significantly higher than that in those without lymph node metastasis. Koay et al^11^ showed that necrosis in GIST indicates poor outcome, and Singer et al^12^ found that epithelioid and mixed cell-type GIST had worse outcome than spindle cell-type GIST. This suggests that mixed GIST and GIST with necrosis may be more aggressive and more prone to lymph node metastasis.

At present, there are few data on gene mutation detection in patients with GIST lymph node metastasis reported in the literature, and most of these are *KIT* exon 11 mutations. This study also showed that half of the GIST patients with lymph node metastasis had *KIT* exon 11 mutations. In addition, half of the SDHB-deficient GIST patients in the study cohort developed lymph node metastases, which is consistent with the results of previous studies.^13^

Previous study has indicated that distant metastasis is an independent risk factor for GIST lymph node metastasis.^9^ This study suggests that the tumor site not located in the stomach, the largest tumor diameter >10 cm, and that distant metastasis is closely related to GIST with lymph node metastasis. Based on these three factors, we constructed a heatmap for lymph node metastasis prediction. The results showed that patients with GIST with a tumor diameter greater than 10 cm and distant metastasis had a higher rate of lymph node metastasis. For such patients, in addition to the treatment of the primary tumor, careful abdominal exploration should be emphasized, and the suspected lymph nodes should be removed as much as possible.

The main route of GIST metastasis is either blood transfer to the liver or direct dissemination through the peritoneum. The status of lymph node metastasis in the natural history of GIST is not yet clear, but some scholars have suggested that lymph node metastasis is a late event in the natural history of GIST, which generally occurs after blood vessel metastasis.^10^ Hou et al^14^ evaluated the relationship among clinicopathological parameters, tumor stage, and outcome in 613 patients with GIST, screened 12 clinicopathological indicators, including lymph node metastasis, and suggested that patients with lymph node metastasis have a poor outcome. Guo et al^8^ included 1024 GIST patients who underwent surgery, of which 198 received lymph node dissection due to lymphadenopathy. Among these, lymph node metastasis was confirmed in 17 patients. After PSM, analysis revealed that patients with lymph node metastasis had significantly worse PFS compared to those without lymph node metastasis. The present study showed that RFS in the group with lymph node metastasis and no distant metastasis was inferior to that of the nonmetastasis group and was comparable to that the group with distant metastasis and no lymph node metastasis, suggesting that lymph node metastasis may be an advanced event in the course of GIST.

The AJCC staging system for GISTs directly classifies patients with GIST with lymph node metastasis as stage IV, which also suggests that GIST lymph node metastasis is an adverse prognostic event during GIST. Imatinib had a satisfactory effect on advanced GIST. Surgery combined with postoperative imatinib therapy has become the main treatment method for preventing or controlling postoperative recurrence and metastasis in high-risk patients with GISTs. Currently, the guideline-recommended suitable population for postoperative adjuvant therapy for GIST is patients with a higher risk of recurrence who have received adjuvant therapy for at least three years.^3^^,^^15^ Given that GIST with lymph node metastasis may indicate that the tumor is more aggressive, and the outcome is comparable to that of the group with distant metastasis, long-term use of targeted drugs in such patients may help improve their long-term survival rates.

This study has some limitations. First, this was a retrospective study with the intrinsic weakness of potential bias. At the same time, although the PSM method was adopted to improve the comparability of the curative effects of the three groups of patients and significantly reduce the selection bias, it could only balance and control the observable confounding factors but could not control for unobservable factors. Second, this study exclusively included patients who presented with lymphadenopathy identified either through preoperative imaging or intraoperative exploration and subsequently underwent lymph node dissection or lymphadenectomy. Therefore, the lymph node metastasis rate may have been overestimated in this study. Third, Due to the limited number of cases in the group with concurrent lymph node and distant metastases, this subgroup was excluded from subsequent prognostic analyses.

In conclusion, this multicenter study, utilizing PSM on a large-scale dataset, identified tumor location, tumor diameter, and distant metastasis as independent risk factors for lymph node metastasis in GIST. Patients with lymph node metastasis exhibited significantly inferior RFS compared to those without metastasis, and comparable RFS to those with distant metastasis. These findings suggest that lymph node metastasis is an advanced event in GIST, which hold significant clinical implications for the diagnosis and management of GIST patients with lymph node metastasis.

## Data Availability

The data are considered personally identifiable, although all personal identifiers have been removed before data-cleaning and analyses. Therefore, the database cannot be shared publicly. And, the data can be made available to researchers upon reasonable request, in the condition of clearance by the responsible ethics committee.
